# Potential path volume (PPV): a geometric estimator for space use in 3D

**DOI:** 10.1186/s40462-019-0158-4

**Published:** 2019-04-29

**Authors:** Urška Demšar, Jed A. Long

**Affiliations:** 1 School of Geography & Sustainable Development, University of St Andrews, Irvine Building, North Street, Scotland, St Andrews KY16 9AL UK; 2 Department of Geography, Western University, London, ON Canada

**Keywords:** Movement analytics, Animal movement, 3D, Time geography, Space use, Volumetric visualisation

## Abstract

**Background:**

Many animals move in three dimensions and many animal tracking studies collect the data on their movement in three physical dimensions. However, there is a lack of approaches that consider the vertical dimension when estimating animal space use, which is problematic, as this can lead to mistakes in quantification of spatial differentiation, level of interaction between individuals or species, and the use of resources at different vertical levels.

**Methods:**

This paper introduces a new geometric estimator for space use in 3D, the Potential Path Volume (PPV). The concept is based on time geography and generalises the accessibility measure, the Potential Path Area (PPA) into three dimensions. We derive the PPV mathematically and present an algorithm for their calculation.

**Results:**

We demonstrate the use of the PPV in a case study using an open data set of 3D bird tracking data. We also calculate the size of the PPV to see how this corresponds to trip type (specifically, we calculate PPV sizes for departure/return foraging trips from/to a colony) and evaluate the effect of the temporal sampling on the PPV size. PPV sizes increase with the increased temporal resolution, but we do not see the expected pattern than return PPV should be smaller than departure PPV. We further discuss the problem of different speeds in vertical and horizontal directions that are typical for animal movement and to address this rescale the PPV with the ratio of the two speeds.

**Conclusions:**

The PPV method represents a new tool for space use analysis in movement ecology where object movement occurs in three dimensions, and one which can be extended to numerous different application areas.

**Trial registration:**

N/A

**Electronic supplementary material:**

The online version of this article (10.1186/s40462-019-0158-4) contains supplementary material, which is available to authorized users.

## Introduction & Background

Many animals move freely in all dimensions within the biome and so the most appropriate characterisation of their space use and the patterns in their movement should use all three physical dimensions [1]. Of these, the first two represent the surface of the Earth and are either represented as geographic coordinates (longitude, latitude) or projected in some type of projected coordinate system (x – northing, y – easting). The third coordinate differs depending on the animal species: it can be elevation for species moving in highly variable terrain, such as deer in the mountains [2], altitude above the surface of the Earth for flying species [3] and species with a habitat in the forest canopies [4] or depth for fish [5, 6] or fossorial species [7].

Regardless of tracking technology used, data on animal movement are often mathematically represented in the form of trajectories, that is, sequences of geographic locations sampled at regular or irregular times. Many current tracking technologies allow location sampling in three physical dimensions: for example, GPS trackers measure elevation as well as longitude and latitude (although with worse accuracy for elevation than for the two surface dimensions) [8] and many bio-loggers for marine species include depth sensors [9]. Three-dimensional location can also be extracted from stereo video footage [6] or in recent studies sometimes using passive integrated transponder tracking [7].

Despite the increased availability of three-dimensional location in animal tracking data, the majority of space use methods are still based on only two geographical dimensions. In some cases the third dimension is independently examined, but in general it is altogether ignored. One of the most important concepts in ecology is the ecological niche hypervolume, which is the area within a multi-dimensional resource space that supports a species to persist and reproduce [10]. The most important dimensions of the niche are the three physical spatial dimensions and spatial differentiation in this 3D space is one of the vital factors of preventing competition among species [11]. Other dimensions (e.g. food resources or time use) are complementary to space use and can overlap even if there is spatial differentiations of niches. This differentiation often occurs through differences in use of vertical space [1] and yet the predominant way to explore such differentiations is by reverting to the two geographic dimensions only. This is problematic, since two-dimensional approaches overlook the vertical space use, which, for species with a strong vertical component in their movement means that estimates of typical characteristics, such as the size of the home ranges, the amount of spatial overlap and consequently the level of interaction between individuals, and the use of resources at different vertical ranges, are often incorrect [12].

The reasons for third dimension being widely ignored in ecology are not very clear, especially since multi-dimensional statistical methods have existed for some time [1]. One reason could be the historically relatively high inaccuracy of vertical measurements in GPS trackers, which are one of the main sources of 3D data in ecology. This problem may soon be resolved with the deployment of the European positioning system Galileo [13] and the Chinese system COMPASS/Beidou, both of which are expected to significantly improve the accuracy of elevation measurements [14]. The second reason applies to marine tracking, where the third dimension, i.e. the depth, is typically collected by a different tag than the two geographic dimensions due to difficulties of satellite localisation for animals that live in water. Depth is normally collected using one of the triple combination data loggers (e.g. Conductivity-Temperature-Depth or a Time-Temperature-Depth), while the 2D location comes from GPS or ARGOS tags for animals that frequently surface, such as sea mammals or from various types of acoustic localisation for fish. Linking depth and localisation data therefore requires further processing, such as dead-reckoning between 2D locations and temporal data linkage, which can be a complex process with a high uncertainty [15]. The third reason may be that including the third dimension into movement models results in more complex geometrical calculations and introduces both a higher computational cost and a potentially more difficult interpretation of results. However, three-dimensional data analysis methods are common in other fields, including areas where using the full range of the three dimensions in data is inevitable, such as 3D information visualisation of volumetric medical data [16]. These methods frequently employ geometric tools to optimise the complexity of 3D methods [17].

Some recent 3D space use studies introduced variations of kernel density estimates (KDE) in three dimensions and compared them with their two-dimensional equivalents [6, 7, 18]. Others also incorporated time into their KDE methods, through development of new spatio-temporal kernels [19] or through adaptation of existing methods, such as for example an extension of Brownian bridges [20] into four dimensions [21, 22]. Outside of ecology Zou et al. [23] present a 4D time density algorithm and demonstrate its use on airplane trajectories. However, all these methods come with a high computational cost and are therefore relatively underused.

In this paper we propose a new geometric method for 3D space use, which is computationally fast in comparison with probabilistic methods. To do this, we generalise two well-known time geography concepts, i.e. the *Space-Time Prism* and the *Potential Path Area* [24], into four dimensions (three geographic dimensions and time). Time geography was introduced by Hägerstrand [25] to represent movement of people in a conceptual space of a *Space-Time Cube* (STC), which consists of two geographic dimensions and time on the third axis. In this conceptual space, the volume that can be reached by a moving object given its observed speed is defined as a *Space-Time Prism* [24–26]. The projection of this volume to the two geographic dimensions is an accessibility ellipse, called *the Potential Path Area* (PPA). PPAs are relatively easy and quick to calculate from movement trajectories and were proposed as a geometric estimator of animal space use in two geographic dimensions [27, 28].

We extend the principle of PPA into *Potential Path Volumes* (PPV) and propose that they could be used as a 3D space use estimator for trajectories where location is measured in three dimensions [26]. We define PPVs mathematically, present an algorithm for their calculation and demonstrate how they work on simulated data. We further apply PPVs on a case study using real animal movement data, where we demonstrate the use of PPVs for visualisation of the uncertainty of movement and evaluate how the size of PPVs depends on temporal sampling resolution. In human mobility and transportation research, space-time prisms and PPAs are a common approach for modelling accessibility and are represented in different ways. A volumetric representation of the 2D prism was proposed by Forer [29], while Miller [24] developed a formal analytical definition for n-dimensional space-time prisms. This paper contributes by introducing an algorithm for a volumetric representation of the PPV in the 4D space and an application in the movement ecology context, where this type of modelling hasn’t been known before.

Our method is primarily aimed to represent unconstrained movement in three dimensions and is suitable for animals which live in air or in water. It may be of some value for movement that is loosely bound to three dimensions (such as movement of monkeys in tree canopies [4]), but less appropriate for movement where species are physically bound to the surface, even if this surface is three dimensional and there is an elevation component to the movement. Examples of this are deer climbing mountains [2] or ants crawling within a three-dimensional nest [30]. Deer movement poses a different problem in terms of three dimensionality and may be better suited to be modelled with 2D PPAs superimposed on the terrain and reshaped based on the energy needed to climb in a certain direction. Ant movement has been represented using a network of chambers in the nest [30], which could serve as the frame for a three dimensional flow network, to be analysed with flow methods from human geography, such as spatial interaction modelling [31] or community detection [32].

The rest of this paper is structured as follows: in the Methods section we mathematically define the PPVs, present an algorithm for their calculation and demonstrate its use on simulated 3D correlated random walks. We introduce the case study by describing the data used and the intended use of PPVs. This is followed by the results and we conclude with a discussion where we comment on the potential of further methodological developments for 3D space use, such as consideration of external environmental conditions that affect 3D movement in our model.

## Methods

**Fig. 1:**
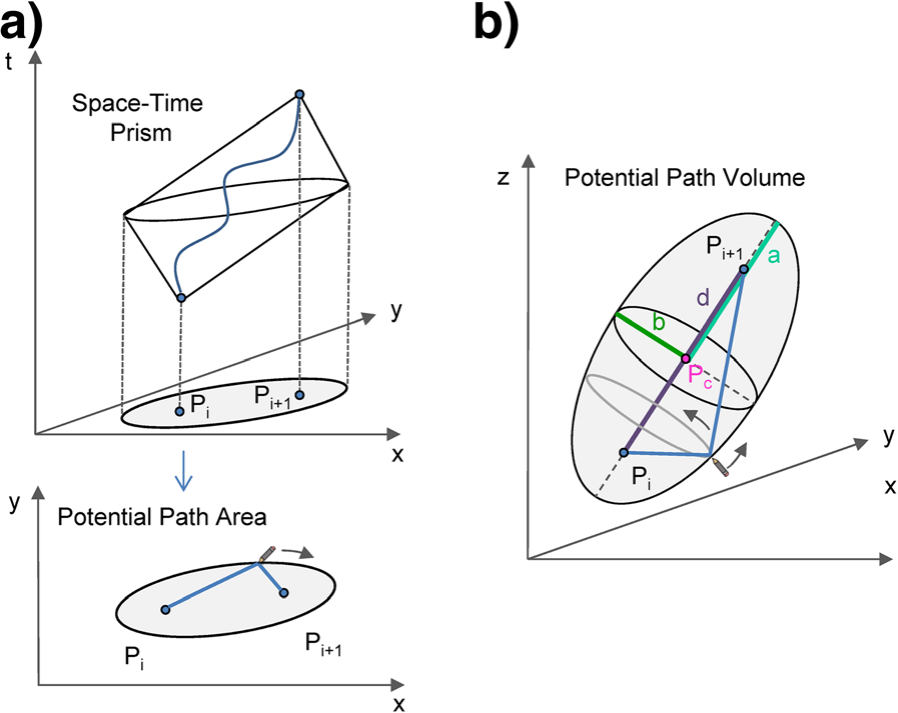
Geometrical derivation of the Potential Path Volume (PPV). **a** The Potential Path Area (PPA) is a projection of the Space-Time Prism onto the two-dimensional movement space and can be generated by tracing the outline of all possible paths between the two movement points *P*_*i*_ and *P*_*i + 1*_. **b** The Potential Path Volume (PPV) is a generalisation of PPA into a three-dimensional movement space and generated in a similar manner by tracing the outline of all possible paths between the two movement points

We generalise a two-dimensional time geography concept, the Potential Path Area (PPA, [24]) into three geographic dimensions. PPA is an accessibility measure from transport geography [33] but has also been used as a geometric estimator of animal home ranges [27]. Mathematically, the PPA is the projection of a Space-Time Prism (STP) onto the geographical plane of the Space-Time Cube (STC) [24]. The STP represents an accessibility volume within the STC which contains all the possible paths between two observed positions, *P*_*i*_ and *P*_*i + 1*_ (Fig. 1a), and whose size and orientation depend on the speed of movement between the two observed positions. Its projection onto the two geographic dimensions is an ellipse, whose size and orientation also depend on the speed between the two observed locations - this ellipse is called the PPA. The union of all PPAs, one built around each segment of a given trajectory, was proposed as a geometric delineator of the home range in two geographic dimensions [27].

We generalise the PPA ellipse into an ellipsoid in the three-dimensional geographic space, which we call the Potential Path Volume (PPV, Fig. 1b). This ellipsoid is the projection of the four-dimensional Space-Time HyperPrism (the 4D accessibility volume between the two observed positions) onto the 3D base space of the Space-Time HyperCube. We calculate PPVs for all segments of a 3D trajectory, that is, a trajectory where location is measured in three geographic dimensions. The union of PPVs for a trajectory is an accessibility volume in the three-dimensional geographic space. That is, given the observed 3D trajectory data and speed on each trajectory segment, the moving object could not have reached any of the locations outside this volume. The volume can consequently be used to delineate the outer boundary of potential space use in three geographic dimensions.

In the rest of this section we provide the mathematical definition of the PPV and describe the algorithm for calculation of PPVs for a set of given trajectories. We further demonstrate how PPVs work on a set of simulated trajectories, generated as 3D correlated random walks.

### Mathematical definition

To obtain the Potential Path Area (PPA) in a two-dimensional movement space, an ellipse is created around a movement segment by placing the start and end points of the trajectory segment, *P*_*i*_ and *P*_*i + 1*_, in the foci of the ellipse. The ellipse equation is then derived using the maximum possible speed of the object and the time difference between *P*_*i*_ and *P*_*i + 1*_. Given this speed and the elapsed time between the two locations, we can calculate the length of the longest path that the object could have traversed. If we imagine this path as a string fixed in *P*_*i*_ and *P*_*i + 1*_, then placing a pen into this string and tracing as far as possible around the two foci while the pen is constrained by the string creates the ellipse that covers all possible paths that the object could have passed between points *P*_*i*_ and *P*_*i + 1*_ (Fig. 1a). This ellipse is thinner if the actual speed between the two points is closer to the maximum speed, and wider if the actual speed is slower. Note that normally PPA calculation assumes that the actual speed between two points is constant, which can be considered as valid when working with data where locations have been sampled with a high temporal frequency.

To create the Potential Path Volume (PPV) around a segment in a three-dimensional movement space, we generalise this process into 3D (Fig. 1b), where *P*_*i*_ and *P*_*i + 1*_ form the foci of an oblique ellipsoid. The longest possible path between *P*_*i*_ and *P*_*i + 1*_ is again calculated using the time difference between the two points and the maximum possible speed of the moving object in three dimensions. We can then create the PPV ellipsoid by first tracing the ellipse in any plane that is parallel to movement direction between *P*_*i*_ and *P*_*i + 1*_ and then rotate the ellipse around the axis represented by the movement direction. This creates a special type of an ellipsoid, a so-called prolate spheroid, where the two minor axes are identical (Fig. 1b). That is, the ellipsoid has three axes: a major axis *a* and two identical minor axes *b*. This model assumes isotropic movement, meaning that movement along the two axes perpendicular to the line *P*_*i*_ to *P*_*i + 1*_ is equally possible. In a more general case, where the resistance to movement in different directions away from the *P*_*i*_ to *P*_*i + 1*_ line varies anisotropically, the PPV model could use a tri-axial scalene ellipsoid, where all three axes are different (i.e. an ellipsoid with a major axis *a* and two minor axes *b* and *c*, where *b ≠ c*). As with PPA, the assumption is that the actual speed between the two points is constant, which creates a thinner/wider ellipsoid depending on how close the actual speed is to the maximum speed.

**Fig. 2:**
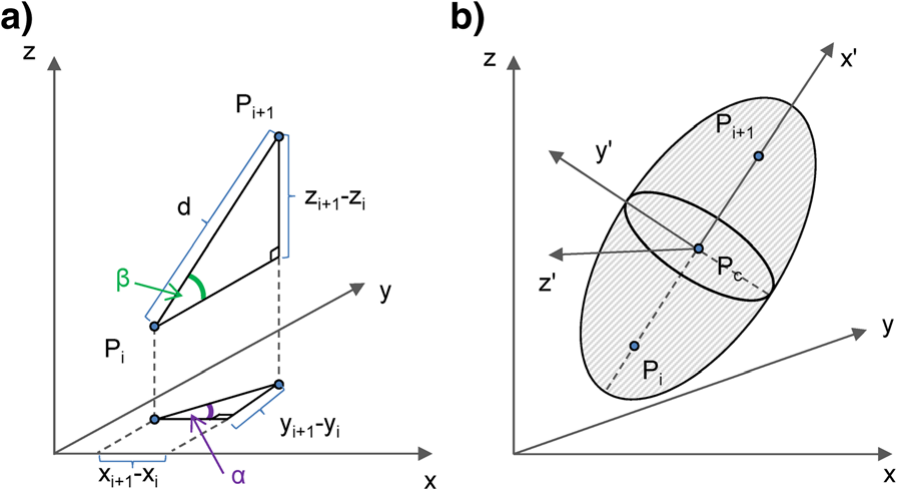
Coordinate system transformation. **a** The definition of rotation angles *α* and *ß* and **b** the transformation of the original data coordinate system *(x, y, z)* into the coordinate system of the ellipsoid *(x’,y’,z’)*

A PPV around each trajectory segment can be generated by knowing the following quantities (Fig. 1b): *d* - the distance between the two foci *P*_*i*_ to *P*_*i + 1*_*, a* – the length of the major axis, *b* – the length of the two minor axes, *P*_*c*_ – the origin point of the ellipsoid’s own coordinate system and the centre of the ellipsoid and the two rotation angles (*α, ß*), which transform the coordinate system of the data into the ellipsoid’s coordinate system (Fig. 2a). In the following we derive each of these quantities.

Given a trajectory segment with start and end points *P*_*i*_*(x*_*i*_*, y*_*i*_*, z*_*i*_*)* and *P*_*i + 1*_*(x*_*i + 1*_*, y*_*i + 1*_*, z*_*i + 1*_*)*, the origin point *P*_*c*_ is the central point between the two end points and is given by:

1 $$ {P}_c=\left({x}_c,{y}_c,{z}_c\right)=\left(\frac{x_i+{x}_{i+1}}{2},\frac{y_i+{y}_{i+1}}{2},\frac{z_i+{z}_{i+1}}{2}\right) $$

We calculate the distance *d* between *P*_*i*_ and *P*_*i + 1*_ as Euclidean distance in a three-dimensional space:

2 $$ d=\sqrt{{\left({x}_{i+1}-{x}_i\right)}^2+{\left({y}_{i+1}-{y}_i\right)}^2+{\left({z}_{i+1}-{z}_i\right)}^2} $$

Knowing the distance *d*, the time difference *Δt* between *P*_*i*_ and *P*_*i + 1*_ and the maximum possible speed of the moving object *v*_*max*_, we can calculate the major and minor axes of the ellipsoid, *a* and *b*. The value of *v*_*max*_ can be either the maximum measured speed in the data, however note that that creates a degenerate ellipsoid on the segment where this speed was observed. We therefore use a more robust model for the maximum speed [27] and calculate it as per this:

3 $$ {v}_{\mathrm{max}}=2.{v}_m-{v}_{m-1} $$

Here *v*_*m*_ is the maximum observed speed and *v*_*m-1*_ is the next largest observed speed. Then, *a* and *b* are calculated using:

4 $$ a=\frac{v_{\mathrm{max}}\cdot \Delta t}{2} $$

5 $$ b=\sqrt{a^2-\frac{d^2}{4}} $$

The final task is to find the equations of the transformation of the original coordinate system *(x, y, z)* onto the ellipsoid axes *(x’, y’, z’)* (Fig. 2b). This transformation is a combination of a translation of the coordinate origin onto the central point *P*_*c*_, followed by two rotations. The first rotation is around the z axis for the angle *α* and the second one around the rotated y-axis for angle *ß* (Fig. 2a). In navigation, *α* and *ß* are the Tait–Bryan nautical angles of pitch and yaw respectively (because of the symmetry of movement around the axis *P*_*i*_ to *P*_*i + 1*_, the third nautical angle, the roll, is not important in our case) and can be calculated as:

6 $$ \alpha =\arctan \left(\frac{y_{i+1}-{y}_i}{x_{i+1}-{x}_i}\right),\ss =\arcsin \left(\frac{z_{i+1}-{z}_i}{d}\right) $$

Once we know the angles and the central point, then the transformation into the new coordinates is defined as follows:

7 $$ \left(\begin{array}{c}{x}^{'}\\ {}{y}^{'}\\ {}{z}^{'}\end{array}\right)=R\left(\ss \right)\cdot R\left(\alpha \right)\cdot \left(\begin{array}{c}x-{x}_c\\ {}y-{y}_c\\ {}z-{z}_c\end{array}\right) $$

where *R(α)* and *R(ß)* are the two rotation matrices given as:

8 $$ R\left(\alpha \right)=\left(\begin{array}{ccc}\cos \alpha & -\sin \alpha & 0\\ {}\sin \alpha & \cos \alpha & 0\\ {}0& 0& 1\end{array}\right),R\left(\ss \right)=\left(\begin{array}{ccc}\cos \ss & 0& -\sin \ss \\ {}0& 1& 0\\ {}\sin \ss & 0& \cos \ss \end{array}\right) $$

The order of rotations in Eq. (7) corresponds to the right to left order in the matrix product, i.e. *α* first, then *ß*. Any point *(x,y,z)* in the original coordinate system is then within the PPV ellipsoid when the transformed coordinates satisfy the following inequality:

9 $$ \frac{{x^{'}}^2}{a^2}+\frac{{y^{'}}^2}{b^2}+\frac{{z^{'}}^2}{b^2}\le 1 $$

### Algorithm to calculate PPVs around a trajectory

So far our solution for PPVs was presented analytically, but for the actual implementation of this solution we discretize the 3D space into a volume (a 3D grid with voxels). The main reason for this is the simplicity of combining several PPVs into one in a necessary step when we expand the segment-based calculations from the previous section into a trajectory-based PPV. While an analytical solution of a trajectory-based PPV is possible (as a polysurface consisting of a union of individual segment ellipsoids), such a description would be limited in terms of visualisation and the possibility of combining the result with other data. Discretising the PPV on the other hand allows for a simple per-voxel combination with other data (both in the algorithm when we calculate the union of PPVs for all segments on a specific trajectory or if PPVs are derived for several individuals and merged into a population-wide descriptor of the use of 3D space, as is commonly done in ecology in 2D). Further, using a volumetric representation, the visualisation of results can be done using standard volumetric visualisation software, such as Voxler or ParaView. Based on all this we have chosen to represent the PPV as a volume.

**Fig. 3:**
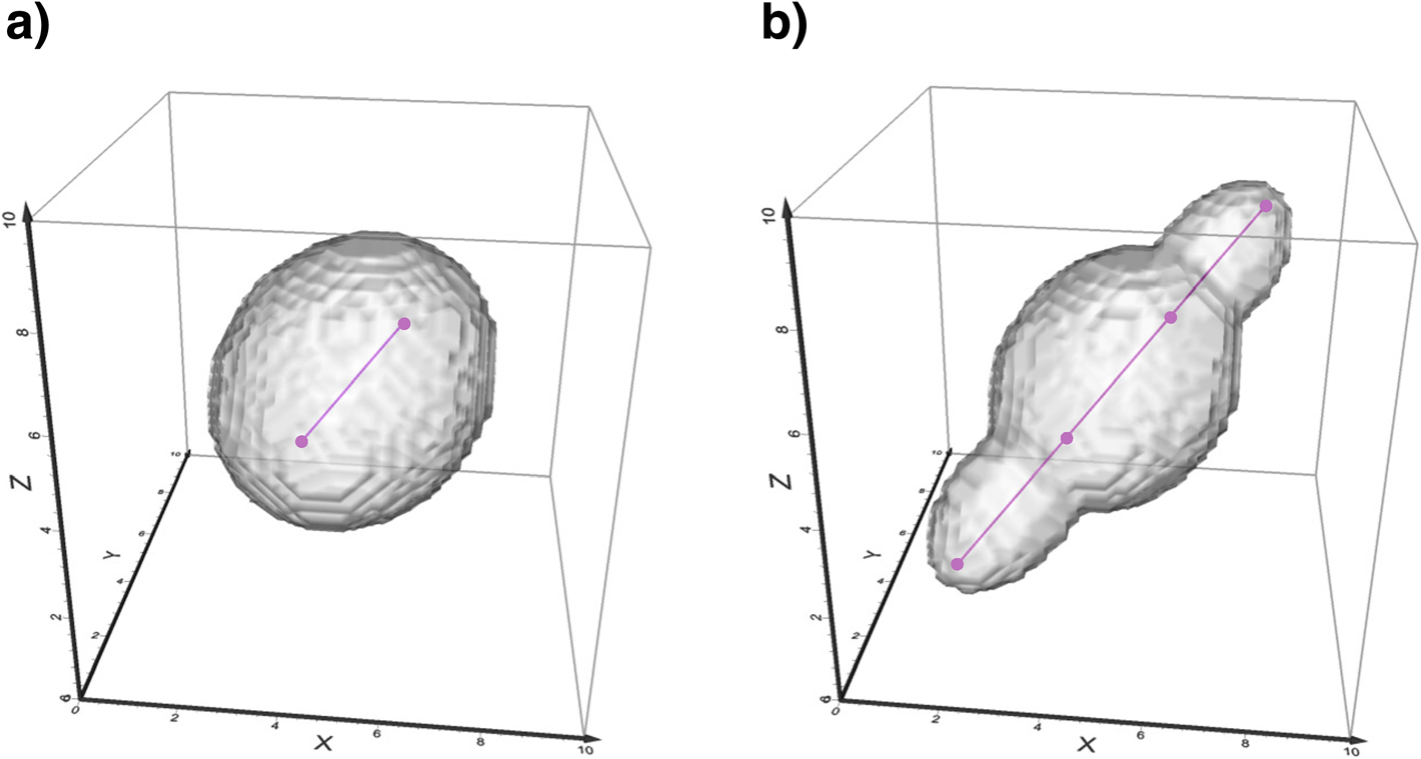
Calculation of the Potential Path Volume. The PPV is shown (**a**) around one trajectory segment and (**b**) around three segments, on which the speed on the first and last segment is higher than on the middle segment. This creates a wider PPV ellipsoid around the slower segment and narrower PPV ellipsoids around the faster segments

The PPV for a trajectory is calculated per segment. For each segment we first find the characteristic values of the PPV ellipsoid (Eqs. 1, 2, 3, 4, 5, 6). To transform the PPV equations into a useable shape we use a discretization process where we divide the study area into small 3D pixels, termed *voxels*. We then transform the coordinates of the centre of each voxel in the PPV volume into the ellipsoid coordinate system (Eq. 7, Eq. 8) and use Eq. 9 to determine if the voxel is inside or outside the ellipsoid. We assign value 1 to internal voxels and value 0 to external voxels to create a Boolean volumetric representation of the PPV ellipsoid around this particular segment (Fig. 3a). The PPV volume for the entire trajectory is then built as a union of PPV volumes for each segment (Fig. 3b). The resulting volume can be visualised using standard volumetric visualisation techniques, such as volumetric rendering, surface mesh, or isosurfaces [34].

The pseudocode for our algorithm is provided in the Additional file 1, while Additional file 2 presents an example of use. The method has been integrated in the *wildlifeTG* R package (available at https://github.com/jedalong/wildlifeTG) and the R code used to generate the PPVs is also available individually at: http://github.com/udemsar/PPV. The code returns a volumetric data set, i.e. a set of voxel coordinates with the value of the PPV in each voxel, which should ideally be visualised in a volumetric visualisation software. We used Voxler (a 3D geology visualisation software from Golden Software) for this purpose, but a similar Free and Open Source Software option is ParaView.

### PPVs on simulated trajectories

**Fig. 4:**
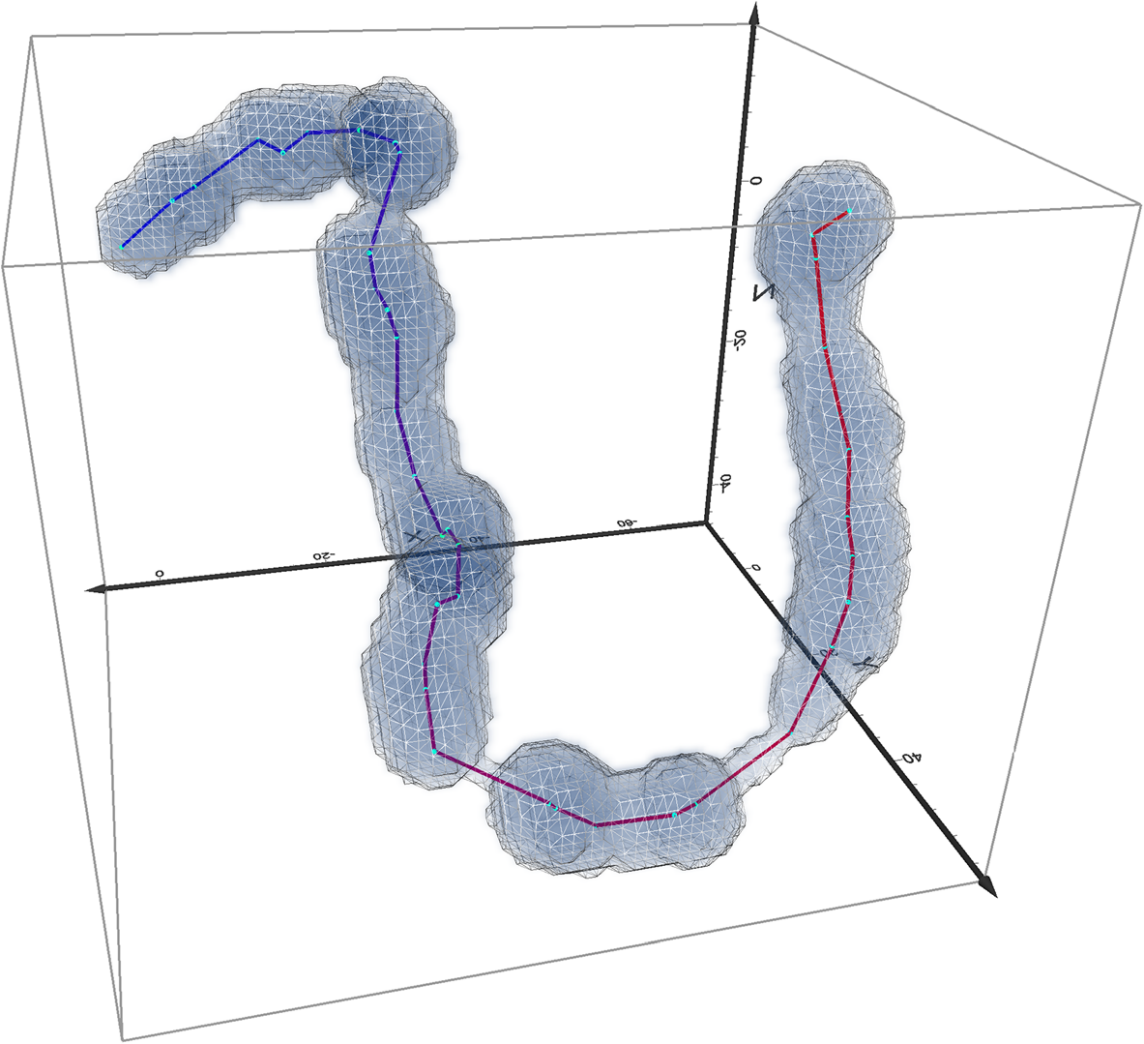
PPV around a 3D CRW. The random walk consists of 40 uniform time steps, shown from red (time = 0) to blue (time = 39). The PPV is shown with a mesh isosurface and volumetric rendering. Note narrower and wider ellipsoids on segments with higher and lower speed respectively

To demonstrate how the algorithm works for movement data, we calculate PPVs around a simulated trajectory, created as a correlated random walk (CRW) in three dimensions. Correlated random walks are commonly used as animal movement models [35]. Steps in a two-dimensional CRW follow similar directional orientations, since most animals tend to keep the general direction of movement at a short temporal scale (i.e. moving forward). This is mathematically described by a small change in the angular direction of movement in the 2D plane from one step in the walk to another. Similarly, we generate a three-dimensional CRW by varying two angular components of movement: the horizontal turning angle that describes the change in direction in the 2D geographic plane and the vertical turning angle that describes change in the vertical direction. We generate the 3D CRW on a series of uniform time steps and different speeds of movement at each step, in order to create a trajectory useful for demonstrating the PPV calculation. Figure 4 shows an example 3D CRW with the respective PPV volume, shown with a surface mesh and volumetric rendering.

## Case study

In this case study we calculate the PPVs on a set of real 3D bird trajectories to demonstrate the potential of the method and investigate the effect of temporal sampling frequency and trip type on PPV size. In the second part of the case study we consider the problem of unequal movement characteristics in horizontal and vertical dimensions and propose a PPV solution where we rescale the coordinates in z direction according to ratio of vertical and horizontal speeds.

### Data

**Fig. 5:**
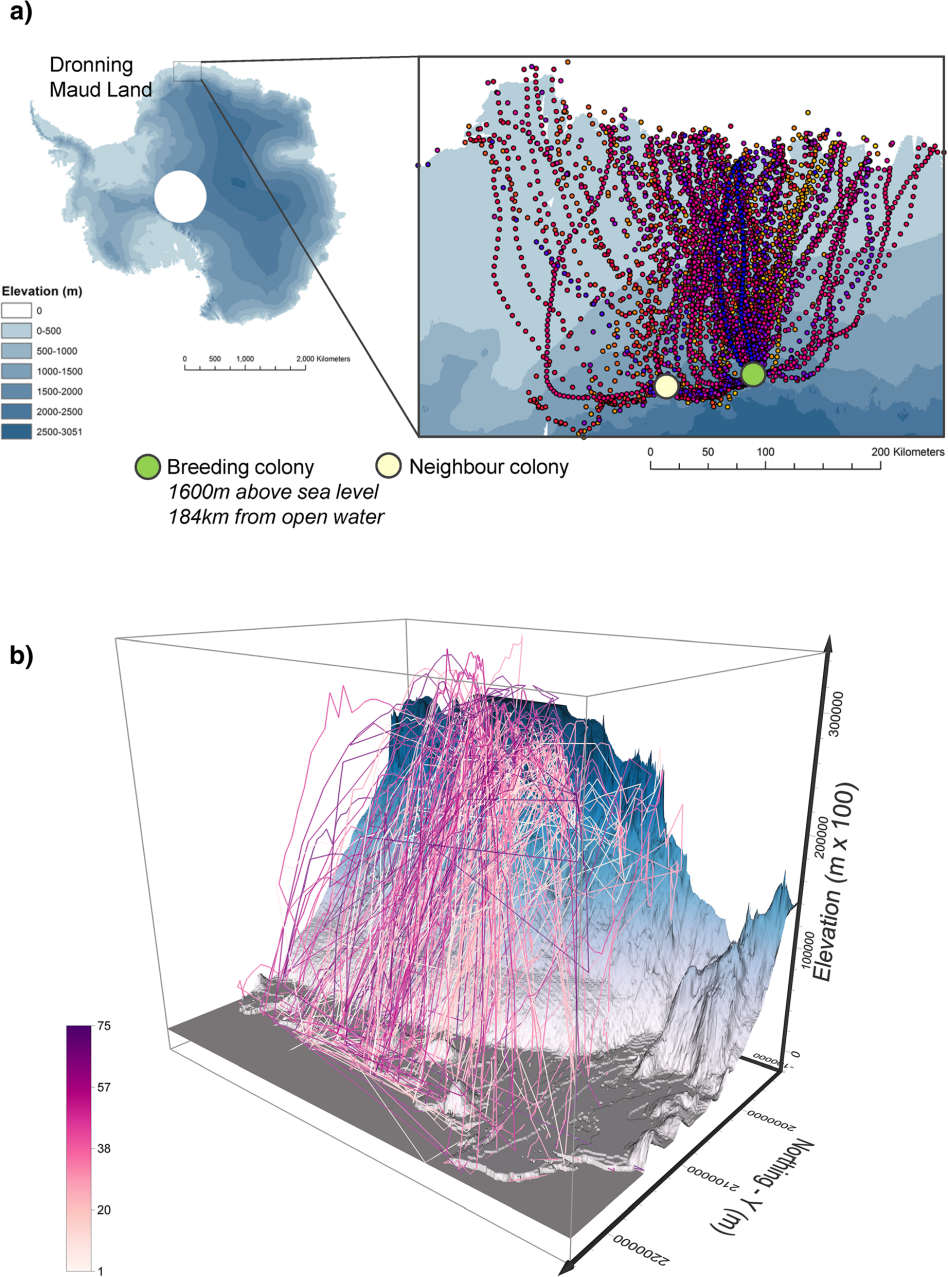
Trajectories of 75 Antarctic petrels (*Thalassoica antarctica*) on return and departure foraging trips [36]. Trajectories are shown over the Digital Elevation Model of Antarctica [37]. Panel (**a**) shows the location of the colonies on Antarctic continent, while panel (**b**) shows trajectories and DEM in real 3D, with a vertical exaggeration of 100 times in order to visually better show movement in three physical dimensions

We used open 3D bird tracking data from a study on avian movement [3], freely available from the Movebank repository [36]. The data consisted of GPS trajectories of foraging trips of 75 individual Antarctic petrels, *Thalassoica antartica*, which were collected to investigate the effect of wind conditions on the avian movement. A full description of the data can be found in Tarroux et al. [3], but here we summarise the points that are relevant to our method. Tracked individuals were tagged in the Svarthamaren breeding colony (71°53′S, 5°10′E) in Dronning Maud Land in Antarctica. The colony is located at 1600 m a.s.l. and 184 km away from the nearest point of (potentially) open water, where petrels can forage, with virtually no opportunities for finding the food in the areas between the colony and the open ocean. Trips were collected during three breeding seasons between 2011 and 2014, where each individual was fitted with a GPS tag and re-captured after returning from one foraging trip. GPS trackers were programmed to capture location at different temporal frequencies: 5 min, 10 min, 30 min, 60 min and 90 min. The mean absolute altitudinal error for GPS data was 52 m [3]. Figure 5 shows the 75 trajectories used in our study, superimposed over a digital elevation model of Antarctica [37] in three dimensions with exaggerated vertical dimension. Data were projected using Polar Stereographic projection with the standard parallel at 70°S.

### The effect of temporal sampling on calculation of PPVs

One of the hypotheses in the Tarroux et al. [3] study is that birds drift more in their departure trips from the colony to the foraging grounds and take a more direct course on their return trips, compensating more strongly for wind drift. While the original study uses GPS tracking data combined with environmental data (atmospheric wind models) to investigate this hypothesis, we explored how this expected pattern would look in a geometric accessibility estimator, such as the PPV. We expected that the larger uncertainty in departure trips vs. return trips would mean that return PPVs should be overall smaller in size than the departure PPVs. Further, the size of the PPVs was expected to increase with the temporal sampling resolution, as longer sampling times produce longer segments, which in turn generates larger ellipses, that reflect larger uncertainty in movement between two observed points.

To test the effect of temporal sampling resolution on the size of the PPV, we separated bird trajectories into three groups with resolution of 5, 10 and 30 min and within each group into departure and return trips. We excluded trajectories with 90 min sampling as well as those trajectories where there were gaps in data that were of more than three times the resolution. For example, if a trajectory sampled at 5 min had a 16 min gap we excluded this trajectory from consideration. Such gaps could have occurred for a number of reasons (for example the GPS could momentarily lose connection to a required number of satellites), with the consequence that the focal distances of the PPV ellipsoid covering these gaps became very large and the resulting ellipsoids encompass the rest of the PPV. The final data set consisted of 68 trajectories: 18 with 5 min resolution, 34 with 10 min resolution and 16 with 30 min resolution.

We set the upper limit of the volume extent to 4000 m as no bird flew higher than 3170 m and the lower limit to -100 m to account for the GPS error creating seemingly underwater points. We then calculated the sizes of the PPVs and tested the effect of trip type (departure/return) and temporal sampling resolution with a two-way ANOVA.

## Results of the sampling effect study

**Fig. 6:**
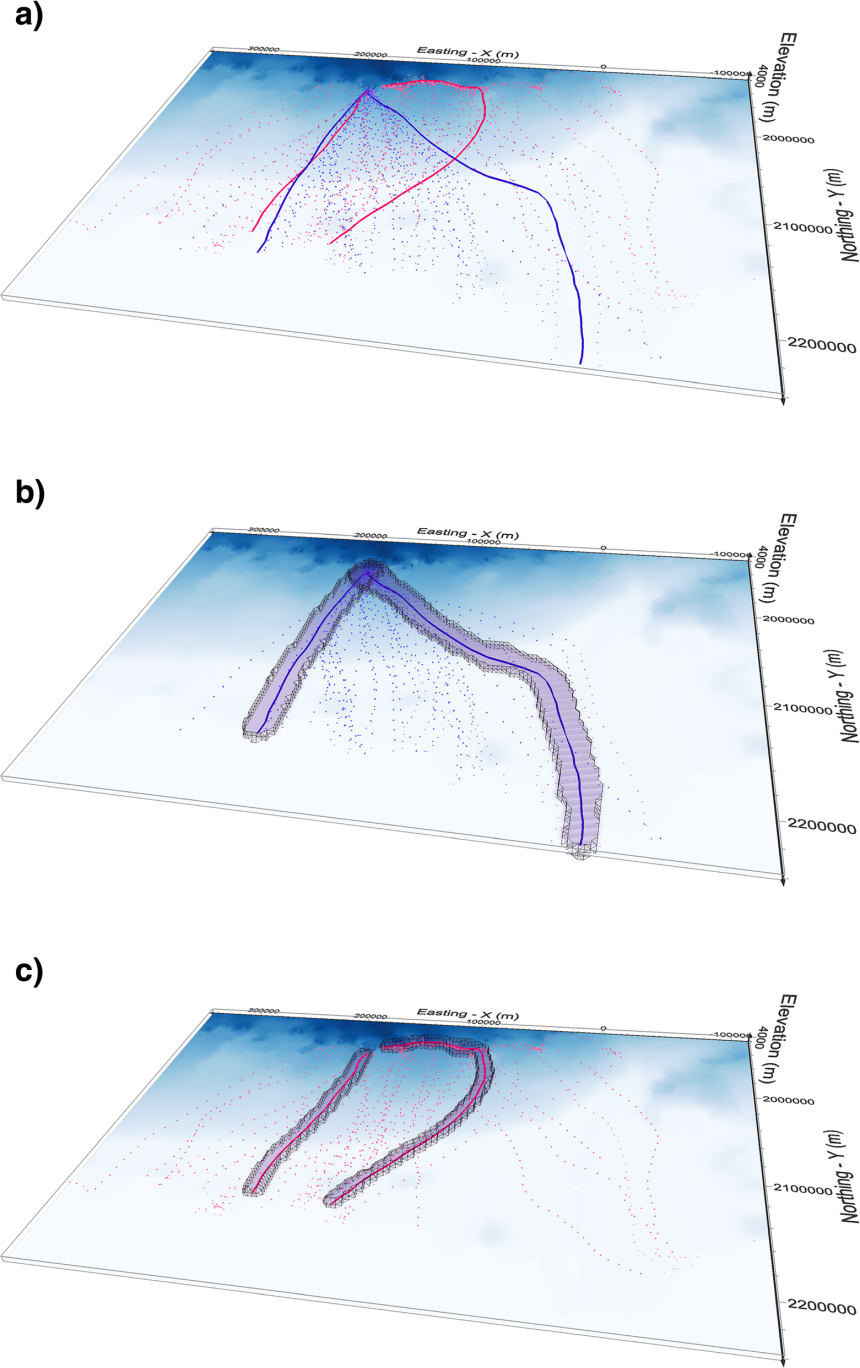
Trajectories and PPVs of two example Antarctic petrels, both with sampling resolution of 10 min. Here PPVs are shown in real 3D (no exaggeration in z dimension). Panel (**a**) shows the departure and return trajectories in blue and pink respectively, where the trajectories of the two selected birds are shown as connected lines. Panel (**b**) is the PPVs of the departure trajectories and panel (**c**) the PPVs of the return trajectories. Note the thinness of the 3D volume when elevation is represented on the real scale and not exaggerated as in Fig. 5

Figure 6 shows trajectories and PPVs of return and departure trips for two example birds. The PPVs in this figure are calculated without vertical exaggeration and are cut at maximum and minimum volume extent as per above.

**Fig. 7:**
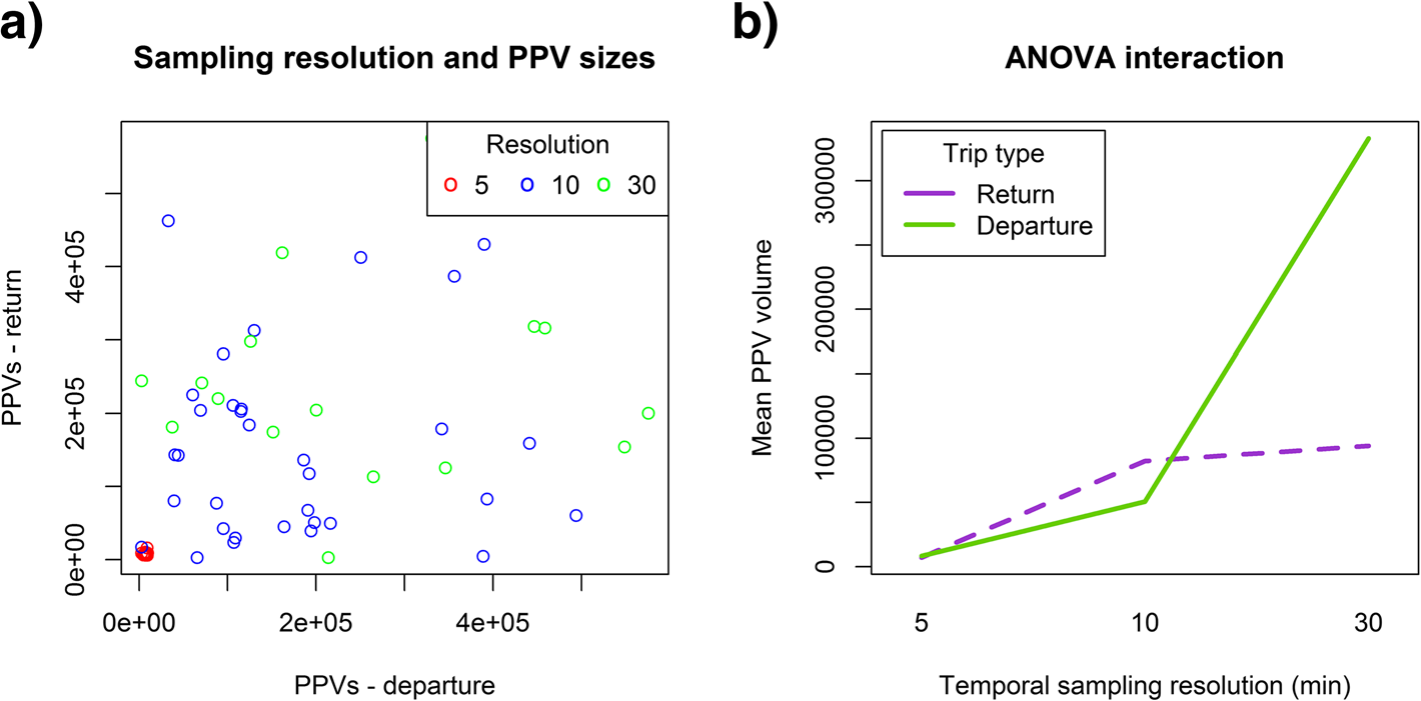
The effect of trip type and temporal resolution on PPV volume sizes. **a** Scatterplots of PPV sizes (in km^3^) for departure and return trips for all three temporal sampling resolutions (in minutes). **b** Interaction plot of ANOVA analysis of PPV volumes vs. temporal sampling resolution and trip types

Table 1 shows average sizes of PPVs for different sampling resolutions and split between departure and return trips. As expected, PPV sizes increase with the increased temporal resolution, but we do not see the expected pattern than return PPVs should be smaller than departure PPVs. This is also not apparent from the scatterplot of departure vs. return trips in Fig. 7a. The results of the ANOVA show that there is a highly significant main effect of trip type (*F* = 33.482, *p* < 0.001), a highly significant main effect of temporal sampling resolution (*F* = 447.679, *p* < 0.001) and a highly significant effect of interaction between the two factors (*F* = 218.309, *p* < 0.001). Figure 7b shows the interaction between trip types and temporal resolution on mean PPV sizes.

**Table 1:** Average sizes of PPVs for departure and return trips and for bird trajectories sampled at different temporal sampling resolutions

Resolution	No. of birds	Trip type	Average PPV (km^3^)	St Dev (km^3^)
5 min	18	Departure	7000	1732
		Return	8324	2361
10 min	34	Departure	82,151	19,538
		Return	50,592	14,932
30 min	16	Departure	93,955	27,688
		Return	333,131	94,857

### Addressing the differences in vertical and horizontal movement

One of the most challenging aspects of estimating the PPV is the selection of an appropriate value for *v*_*max*_. With 2D PPA methods, Long & Nelson [27, 28] suggest several approaches derived from the statistical distribution of the individual segment velocities (i.e., distance / time between points *P*_*i*_ and *P*_*i + 1*_) which we have extended to three-dimensions. The biggest challenge in the estimation of the 2D PPA is the effect of having large and/or varied temporal durations between fixes, which results in a geometric space that overestimates the potential area of movement opportunity. The same effect can occur in the PPV, but is compounded by the fact that in most wildlife applications horizontal movement (i.e., across x and y dimensions) is fundamentally different from vertical movement (i.e., in z dimension). We propose to consider different *v_xy*_*max*_ and *v_z*_*max*_ values for horizontal and vertical speeds, which reflect different movement abilities of individual species.

**Fig. 8:**
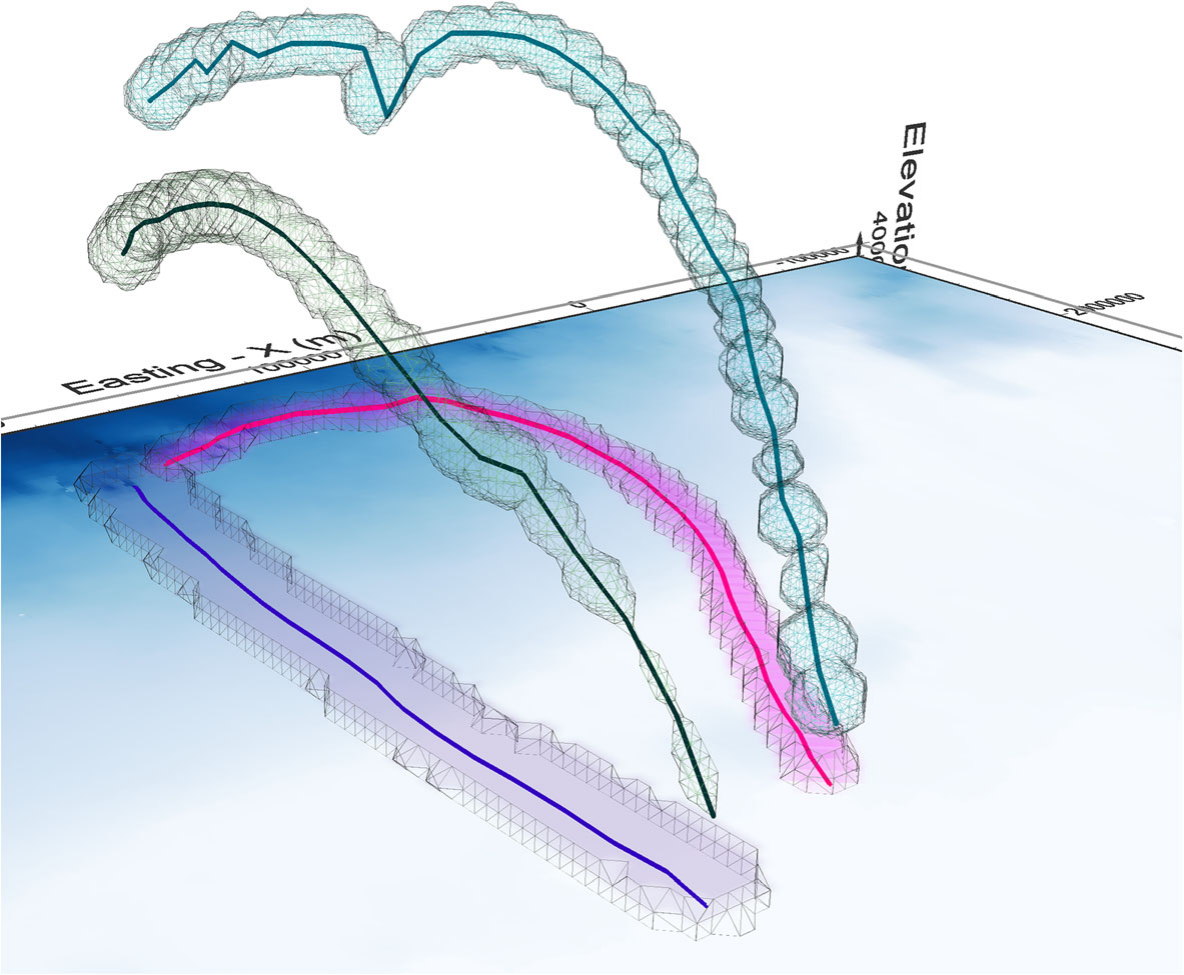
The difference between rescaled and original PPVs. The original trajectories and PPVs are shown in blue (departure trip) and pink (return trip) and are the same as in Fig. 6 – here, the elevation is almost unnoticeable, due to the much larger extent of movement in horizontal than vertical direction. The green and teal trajectories/PPV show the rescaled versions of departure and return respectively, where z dimension of the trajectory coordinates was rescaled (stretched) according to the ratio of maximum horizontal and vertical speeds on each trajectory. This provides a clearer visualisation of the shape of each PPV and allows visual identification of segments with the fastest and slowest movement, corresponding to the thinnest and thickest ellipsoids

Practically, this can be achieved by re-scaling the z-dimension coordinates proportionally to the ratio of *v_z*_*max*_ / *v_xy*_*max*_. If *v_z*_*max*_ < *v_xy*_*max*_ (as would be the case for many species, for example for a long migratory flight at an almost constant elevation) this would have the effect of offsetting the vertical flatness inherent for movement across large distances to make the PPVs effectively flatter or narrower in the vertical dimension. We demonstrate this in Fig. 8, which shows PPVs calculated from the same data as Fig. 6, but where z dimension has been scaled with the ratio of *v_z*_*max*_ / *v_xy*_*max.*_

## Discussion

One of the limitations of our algorithm is that it creates a volumetric representation of movement, which as all volumetric algorithms involves a relatively long processing time. In particular, the computational complexity of our algorithm is O(*n x p x v*), where *n* is the number of trajectories (i.e. the number of moving individuals), *p* the length (number of points) of the longest trajectory and *v* the number of voxels. With high resolution (fine temporal interval) tracking data and representations with fine spatial volumetric resolution (i.e. those where voxels are relatively small), this means that the computational time will be longer and may increase beyond the expectation of what is practical for each dataset. There are ways of getting around this, for example, the complexity could be decreased by at each step considering only voxels from the box that bounds the ellipsoid around every segment instead of the entire volume. Currently the algorithm searches through the entire volume to identify if each voxel is inside the PPV or not. Instead, we could create a pre-cut set of boxes within the volume, which would include only voxels that are nearest to the trajectory, but which can still potentially fall into any of the segment-based ellipsoids. Then the option of being within/out of the PPV could be checked for these voxels only, resulting in fewer calculations than for the whole volume. This kind of improvement for volumetric algorithms is common in computer graphics [17] and has in GIScience been used previously in the case of space-time densities [34].

Conversely, since the PPV defines the maximum volume that the moving object can reach given its maximum speed, it itself could be used to decrease the computational time of other complex three dimensional algorithms for space use. For example, calculations of any of the 3D densities (e.g. [21–23]) could be limited to voxels inside the PPV only, thus reducing the overall time needed for calculation of these volumetric representations.

Another limitation of the proposed model is the isotropic nature of movement in our choice of the ellipsoid. Currently we use a prolate ellipsoid, where the two minor axes are identical, thus assuming the ease of movement being the same in the two directions that are perpendicular to heading. This however is a simplification and a more complex scalene ellipsoid (i.e. the two minor axes are not necessarily the same) would be required to represent anisotropic movement. The anisotropy is related to the energetics and power costs associated with the animals’ physiology, but also the medium which the animal moves through. For example, in the absence of wind or thermoclines a vertical move upwards requires more energy than a comparable one downwards owing to the force of gravity. Vertical movement upward therefore requires higher power costs than downward movement and this could be accounted for by the shape of the ellipsoid. The opposite effects may be found in marine applications where buoyancy forces exceed gravity or where physiological limitations, such as availability of oxygen on deeper dives, may affect movement in vertical direction [15]. Previous movement analytics studies have included kinematics to create physically realistic space-time prisms [38, 39] and a similar approach could be developed to account for energetics and power consumption of animal movement in 3D.

The calculation of the PPV is relevant only in cases where animals exhibit substantial movement in the vertical dimension (as well as horizontal). In many species, even substantial vertical movements will be an order of magnitude less than in the horizontal dimension [15, 40] reflecting different movement physiology and motivations in the vertical direction. We have suggested an approach to rescaling vertical coordinates to account for this effect (e.g., when *v_z*_*max*_ < *v_xy*_*max*_) in an effort to capture differing movement ability in the vertical dimension. However, the temporal resolution of movement in the vertical dimension must also be carefully considered. That is, a fix interval suitable for capturing movement behaviour in the horizontal dimension, may be inappropriate (and likely too coarse) for capturing fine-scale movement in the vertical dimension. Thus, when implementing 3D movement models such as the PPV, the temporal resolution of the data must be examined carefully and consider the scale of movement in both horizontal and vertical dimensions. Where temporal fix intervals are coarse relative to movement behaviour in the vertical dimension (which might be typical), the PPV may overestimate potential movement opportunity in the vertical dimension.

Further, our model does not take into account environmental conditions that may affect the movement. To do this, the ellipsoid could be deformed by modelling the forces exerted by the external situation, for example, the uncertainty volume around a bird flying in strong winds could start as an ellipsoid, but the surface of this ellipsoid could then be stretched, twisted and distorted according to the forces exerted by the wind during the time that elapsed between the two observations’ of the bird’s location. Incorporating environmental conditions into 2D time geography has been previously explored [41, 42]), but is yet to be adapted to three dimensional systems.

PPVs could be used for trajectory annotation with 3D environmental data to support context-aware movement analytics. In 2D, trajectory annotation is a common way to link movement data with environmental data: the value of the environmental variable is calculated for the same moment in space and time as a trajectory point and appended to the trajectory point, creating a so-called semantic trajectory [43]. The calculation is typically done using different interpolation methods, for example the nearest neighbour interpolation or the dynamic interpolation in space [44] or various temporal interpolation methods [45]. For 3D environmental data, such as measurements of wind or the geomagnetic field, we propose that given the uncertainty in the movement, instead of trying to interpolate the field values from the nearest locations, we could consider the uncertainty in 3D trajectory annotation itself. The idea is to use the PPV to delineate the accessibility volume in the environmental field observations, interpolate the environmental field values within the PPV volume and normalise the result with the PPV size as it has been done for other quantitative volumetric measures [46]. The result could then be used to annotate the segment between the two 3D points, to create a 3D semantic trajectory and these data could then be used for identification of movement behaviour through trajectory segmentation or data mining methods [47].

## Conclusions

This paper introduced the Potential Path Volume (PPV) as a new geometric estimator of space use in three physical dimensions. We described its mathematical derivation and presented two examples of use: as a visualisation tool for exploring the uncertainty of movement and as an estimator of the size of the uncertainty volume and how that depends on the temporal sampling frequency. Finally, we demonstrate how the PPV method can be used to study targeted questions pertaining to wildlife movement and space use in 3D. The PPV method represents a new tool for space use analysis in movement ecology where object movement occurs in three dimensions, and one which can be extended to numerous different application areas.1,2

## Additional files

Additional file 1: Pseudocode of the PPV algorithm. (PDF 94 kb)

Additional file 2: A worked example as R markdown. (RMD 3 kb)

## Abbreviations

2D: Two dimensional
3D: Three dimensional
ANOVA: Analysis of variance
CRW: Correlated random walk
GPS: Global positioning system
PPA: Potential Path Area
PPV: Potential Path Volume
STC: Space-Time Cube
STP: Space-Time Prism
