# Supplementary material for: Potential path volume (PPV): a geometric estimator for space use in 3D
Source: Mov Ecol. 2019 Apr 29;7:14. doi: 10.1186/s40462-019-0158-4 (PMC6489342; doi:10.1186/s40462-019-0158-4)
Supplement: Supplementary file 1 — Pseudocode of the PPV algorithm. (PDF 94 kb) [file 40462_2019_158_MOESM1_ESM.pdf]

Additional file 1: Pseudocode for the PPV algorithm

```
Input 1: a set of n trajectories with location measured in 3D, the longest with p segments
Input 2: size of the output volume, given by the number of voxels

Start algorithm
Initialise empty total accessibility volume AV of the size given above.
Outer Loop: for each trajectory
        Initialise empty accessibility volume for this trajectory AV_traj

        Inner Loop 1:
        For each segment P_i – P_{i+1}
                Calculate distance d_i from P_i to P_{i+1} (eq. (2))
                Calculate time difference Δt_i=t_{i+1}-t_i
                Calculate velocity on segment v_i=d_i/Δt_i
        End {for each segment}

        Find maximum possible velocity on this trajectory: v_max (eq. (3))

        Inner Loop 2:
        For each segment P_i – P_{i+1}
                Calculate ellipsoid parameters: a, b, P_c (eq. (1,4,5))
                Calculate rotation angles: α, ß (eq. (6))

                Inner Loop 3:
                For each voxel in AV_traj
                        Take coordinates of the centre of the voxel (x,y,z)
                        Transform into new coordinates (x’,y’,z’) (eq. (7,8))
                        If inside ellipsoid (eq. (9)) then
                                AV_traj(voxel)=max(AV_traj(voxel),1)
                End {for each voxel}
        End {for each segment}
        AV = max(AV,AV_traj)

End {for each trajectory}

Return: AV, a 0/1 volume of voxels being outside/inside the union of ellipsoids of all segments of all trajectories
```
